# Sexual dimorphism in mud crabs: a tale of three sympatric *Scylla* species

**DOI:** 10.7717/peerj.10936

**Published:** 2021-04-12

**Authors:** Hanafiah Fazhan, Khor Waiho, Yushinta Fujaya, Nita Rukminasari, Hongyu Ma, Mhd Ikhwanuddin

**Affiliations:** 1 Higher Institution Centre of Excellence (HICoE), Institute of Tropical Aquaculture and Fisheries, Universiti Malaysia Terengganu, Kuala Nerus, Terengganu, Malaysia; 2 STU-UMT Joint Shellfish Research Laboratory, Shantou University, Shantou, China; 3 Guangdong Provincial Key Laboratory of Marine Biotechnology, Shantou University, Shantou, China; 4 Faculty of Marine Science and Fishery, Hasanuddin University, Makassar, Indonesia

**Keywords:** Discriminant function, Morphometric, Morphology, Portunids, Sexual dimorphism, Scylla

## Abstract

Sexual dimorphism is a common phenomenon in the animal kingdom. To test the consistency of sexual dimorphism patterns among sympatric species of the same genus, ten morphometric characteristics of mud crabs *Scylla olivacea*, *S. tranquebarica* and *S. paramamosain* were measured and compared using Discriminant Function Analysis (DFA). The descriptive analysis revealed that in all three species, body size dimensions and cheliped dimensions were significantly larger in males whereas the abdomen width was female-biased. Also, we described a morphological variation (carapace width, CW ≤ CW at spine 8, 8CW) that is unique to *S. olivacea*. Discriminant function analysis revealed that all nine morphometric characteristics were sexually dimorphic in *S. olivacea, S. tranquebarica* (except right cheliped’s merus length, ML) and *S. paramamosain* (except 8CW). The obtained discriminant functions based on the morphometric ratios (with CW as divisor) correctly classified 100% of adults of known sex of all three species. Further, based on the selected body traits, DFA was able to almost completely distinguish males (94%), but not females (74%), among the three *Scylla* species. This study highlights that congeneric species of portunids (e.g., *Scylla* spp.) show similar sexually dimorphic characteristics (body size and secondary sexual characteristics).

## Introduction

Sexual size dimorphism is defined as the phenotypic differences in body dimensions and proportions between males and females (Glucksmann, 1974), and is common among most animal and plant species (Wyman, Stinchcombe & Rowe, 2013). Sexual size dimorphism may result from natural selection, sexual selection (Jones & Ratterman, 2009), or ecological causes including adaptive pressures (De Camargo & De Oliveira, 2012; Sganga, Piana & López Greco, 2016). Male-biased size dimorphism is common in most brachyuran species as it improves the chances of winning in competition (for food and space) (Brockerhoff & McLay, 2005) and in mating (Reading & Backwell, 2007; Waiho et al., 2015; Fazhan et al., 2017b); Meanwhile, larger body sizes in females allow better investment in reproductive output, i.e., higher fecundity (Anderson, 1994). Brachyuran families that exhibit male-biased sexual size dimorphism includes most species of the family Portunidae (Abelló, Pertierra & Reid, 1990), Potamidae (Parvizi et al., 2017), Ocypodidae (Johnson, 2003) and Sesarmidae (Marochi et al., 2019). Female-biased sexual size dimorphism is less common in brachyurans and has only been reported in the brackish-water crab *Deiratonotus kaoriae* Miura, Kawane and Wada 2007 (Camptandriidae) (Kawane et al., 2012) and *Ilyograpsus nodulosus* T. Sakai 1983 (Macrophthalmidae) (Nakayama & Wada, 2015).

Apart from body size dimensions, crustaceans also show marked sexual dimorphism in terms of secondary sexual characteristics. For example, males of most crab species often exhibit larger chelipeds for territorial defence, combat, courting and mating, with the most obvious being those of the fiddler crabs (Bildstein, McDowell & Brisbin, 1989; Tina, Jaroensutasinne & Jaroensutasinee, 2015), while females tend to have broader abdominal pleons for reproductive purposes (Giraldes et al., 2016; Waiho, Fazhan & Ikhwanuddin, 2016). These phenotypic differences between sexes greatly influenced the commercial value of economically important brachyuran species. For instance, in mud crab *Scylla* De Haan 1833, larger male crabs with prominent secondary sexual characteristics and females with well-developed ovaries are high-priced and highly sought-after by consumers (Waiho et al., 2016; Tang et al., 2020).

Although some sexual dimorphic characteristics are obvious, often most characteristics are differentiated quantitative and qualitatively via statistical analysis based on comparative morphometric techniques (Bertin et al., 2002) to allow precise differentiation of sexually dimorphic characteristics based on sex (Nakayama & Wada, 2015), life stages (Parvizi et al., 2017) or species (Barría et al., 2014). Owing to their hard exoskeleton and the presence of spines in many parts of their body, the measurement of morphometric characteristics in crustaceans are much easier to be conducted, making them a suitable model for morphometric studies (Hartnoll, 1982). Analysis of morphometric characteristics in crustaceans is useful in taxonomic, ecological, behavioural and evolutionary studies (Roth & Mercer, 2000; Accioly et al., 2013). For example, morphometric and molecular comparisons revealed that the smaller subtidal *Cyrtograpsus affinis* (Dana 1851) is closely related to the larger intertidal *C. altimanus* Rathbun 1914, and that they are postulated to be of the same species with high ecological and phenotypic plasticity (Spivak & Schubart, 2003). Further, morphometric variation is also useful for fisheries stock management (Bissaro, Gomes-Jr & Di Beneditto, 2012) as geographic variation in morphometry exists among stocks (Cadrin, 2000). The discriminant functions constructed using the morphometric differences between sacculinid-infected and healthy mud crabs also allow the detection of infected individuals based on their reduced morphometric characteristics (Waiho et al., 2017).

Discriminant function analysis categorises individuals to a certain group based on the linear combination of selected predictor variables. This method has been used in species identification (Jaiswara, Nandi & Balakrishnan, 2013), sex determination (Dechaume-Moncharmont, Monceau & Cezilly, 2011; Ferrer et al., 2016) and in the study of sexual size or shape dimorphism in various species, including birds (Niemc et al., 2018), fish (McGrath & Hilton, 2012), shrimps (Mantel & Dudgeon, 2005; Christodoulou & Anastasiadou, 2017) and crabs (Alencar et al., 2014; Parvizi et al., 2017). A thorough understanding of the body shape difference between sexes, especially in crustaceans provide important information regarding its physiological needs (Hartnoll, 2006; Waiho et al., 2016), ecological importance (Simpson, Ambrosio & Baeza, 2016) and evolutionary trends (Rufino, Abelló & Yule, 2004).

Mud crabs belonging to the genus *Scylla* are economically important crustacean species widely found throughout the Indo-West-Pacific region (Waiho et al., 2018; Fazhan et al., 2020). The global capture and aquaculture production of *Scylla* increased steadily within the last two decades and reached 45,393 t and 89,390 t in 2016, respectively (FAO, 2020). There are currently four species within this genus, i.e., *S. serrata* (Forskål 1775)*, S. tranquebarica* (Fabricius 1798)*, S. paramamosain* Estampador 1950 and *S. olivacea* (Herbst 1796) (Keenan, Davie & Mann, 1998), of which only the latter three species live in sympatry in the equatorial region around the South China Sea (Fazhan et al., 2017a; Fazhan et al., 2021). The taxonomic history of *Scylla* was eventful, with some researchers regarded all four as a single species (Stephenson & Campbell, 1960) while some disagreed (Estampador, 1949; Serene, 1952). Keenan, Davie & Mann (1998) revised the taxonomic classification of *Scylla* and classified this genus into four distinct species based on their morphological, morphometric and molecular variations. However, the morphological variation among species described by Keenan, Davie & Mann (1998) focused primarily on male specimens. To date, not much attention has been given to characterise the sexual size dimorphism characteristics between sexes and among species of *Scylla*. The current understanding of the sexual size dimorphism of *Scylla* spp.—males are generally larger—are based on observations and local knowledge. The lack of concrete data in this area greatly hinders the incorporation of numerous biotechnological manipulation techniques associated with commercially important traits, especially body size (Mei & Gui, 2015), into the enhancement of mud crab production (Shi et al., 2019; Waiho et al., 2019). For example, the characterisation of sexually dimorphic characteristics would allow better mapping of valuable traits using high-density linkage map, aid in the selection of potential regulatory genes and the identification of sex-specific markers (Waiho et al., 2019).

Therefore, this study aimed to characterise the morphometric variation in terms of body size dimensions and secondary sexual characteristics between males and females of mud crab genus *Scylla*, to identify which morphometric characteristics better reflect the sexual size dimorphism in mud crabs, and to compare these sexual dimorphic variations among species.

## Material and Methods

### Collection and characterisation of samples

Mud crabs were collected using commercial crab traps once every two months from three locations within Malaysia, namely Matang Mangrove Forest Reserve, Perak (4°45′N100°37′E), Setiu Wetlands, Terengganu (5°39′N102°43′E), and Kota Marudu Mangrove Forest, Sabah (6°44′N117°1′E) within the period of approximately three years (April 2012 to March 2015). No specific licensing is needed for mud crab acquisition as all sampling locations are common crab landing sites and mud crabs are not endangered or protected species. In addition to periodical sampling, morphometric measurements of mud crabs were also measured and recorded from the catches of local fishermen crabbing at the same sampling sites. Only healthy individuals with complete appendages were used. All morphometric characteristics of crabs were measured in the field when they were still alive. Crabs were either returned to fishermen, released back to the wild or used in other studies after data collection. To minimise confounding effect of allometric growth and sexual maturity, only mature specimens with carapace width (CW) of more than 95 mm were selected (Waiho et al., 2016). A total of 5,400 mature individuals were randomly sampled, i.e., 1,800 (900 males; 900 females) per species. Species identification was based on the key morphological characteristics provided by Keenan, Davie & Mann (1998), which includes polygonal patterning, coloration of the chelipeds, shape and height of the frontal lobe spines, and the presence and height of carpus and propodus spines on the chelipeds. Crabs were sexed according to their abdomen morphology—pointed and narrowed in males; darkened, globular and wide in mature females (Waiho et al., 2016; Fazhan et al., 2017b).

### Morphometric characteristics measured

**Figure 1:**
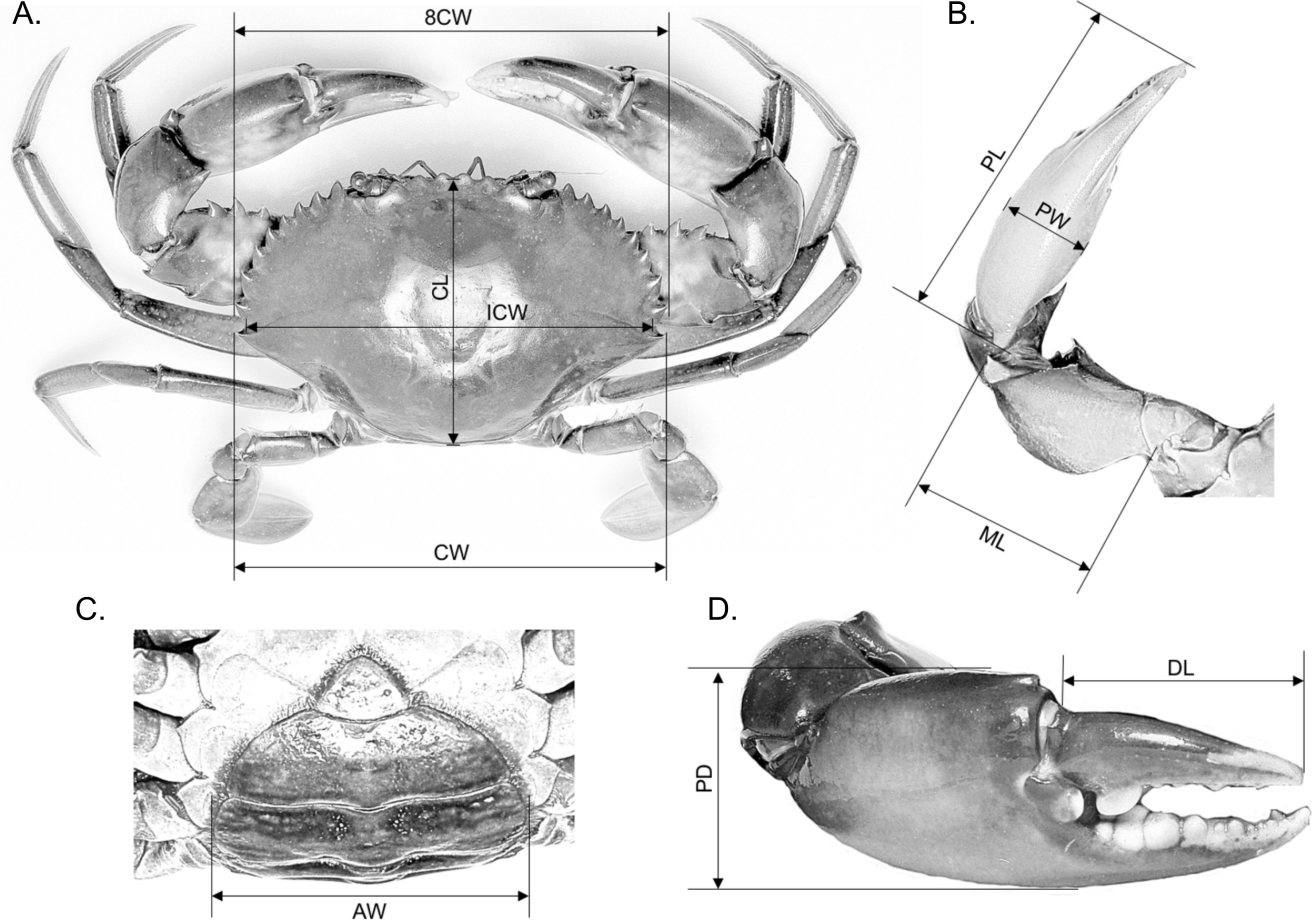
Measurement of various body parts of mud crab *Scylla* spp. used in this study. (A) Overall body dimensions—carapace width (CW), CW at spine 8 (8CW), internal carapace width (ICW) and carapace length (CL). (C) Abdomen width (AW). (B & D) Overall right cheliped dimensions—right cheliped’s propodus length (PL), right cheliped’s propodus width (PW), right cheliped’s merus length (ML), right cheliped’s dactyl length (DL) and right cheliped’s propodus depth (PD).

Ten morphometric characteristics, i.e., CW, CW at spine 8 (8CW), internal carapace width (ICW), carapace length (CL), abdomen width (AW), right cheliped’s dactyl length (DL), right cheliped’s propodus depth (PD), right cheliped’s propodus width (PW), right cheliped’s propodus length (PL) and right cheliped’s merus length (ML) were measured to the nearest 0.01 mm using a standard Vernier calliper (Fig. 1). The AW measured in both males and females referred to the largest width of the fifth abdomen somite. All raw measurements are provided in File S1.

### Statistical analyses

Statistical analyses were conducted using IBM SPSS Statistics version 20. We were only interested in discerning the sexual size dimorphism in the three *Scylla* species; hence variation due to geographical factors and inter-seasonal differences were not included in our analysis and crabs were grouped according to species followed by sex. The exclusion of geographical and inter-seasonal variables enables the generation of functions that could be used to evaluate the differences between sexes of adults caught throughout the year regardless of the sampled location. To assess the assumption of homogeneity of variances, all measurements were subjected to Levene’s test before proceeding to independent sample’s *t*-test to characterise the sexual size dimorphism between sexes in each species. Variables that failed to demonstrate equal variances were analysed using *t*-test with Welch correction. Measurements of abdomen and cheliped dimensions, i.e., AW, DL, PD, PL, ML and PW were converted to ratios by dividing by body size, i.e., CW before comparison between sexes. CW is the standard size measurement used in most portunids (Josileen, 2011), including mud crabs (Keenan, Davie & Mann, 1998; Waiho et al., 2016). By visual observation, the CWs of *S. paramamosain* and *S. tranquebarica* were larger than 8CW, whereas that of *S. olivacea* were smaller than 8CW. Thus, within each *Scylla* species, CW and 8CW were compared using paired *t*-test to validate this observation.

Further, nine morphometric ratios with CW as the divisor (control variable) were tabulated for males and females of each species and subjected to Stepwise discriminant function analysis (DFA). In addition, we also conducted DFA based solely on sex (combined species) to determine if the variation was significant among species. The minimum F value was set as 3.0 (0.05 significance level) (Keenan, Davie & Mann, 1998; Waiho et al., 2017). For functions discerning sex of each species, the halfway point of the two centroid values was used as the cut-off value of the derived function (Waiho et al., 2017). The significance of the derived discriminant function was tested using Wilks’ lamda (U statistic) while the significance of lambda was computed using Bartletts’ V transformation (chi-square statistic). Results were expressed as percentage of correct classification and the robustness of the function was validated using split-sample validation (cross-validation) method found within the SPSS package. For the comparison of morphometric ratios among species, One-way ANOVA with Welch correction and subsequent Games-Howell test were used.

## Results

Comparing the sexual dimorphism of each species separately, the overall size dimensions (CW, ICW, 8CW, CL) of all three *Scylla* species were significantly larger in males than in females (all *P* < 0.01) (Table 1). In addition, secondary sexual characteristics such as AW and cheliped dimensions showed obvious sexual dimorphism as well. The AW of females was significantly larger compared to their male counterparts. Most cheliped dimensions were significantly larger in males, except for PL and ML (all *P* < 0.001) (Table 1).

**Table 1:** The comparison of morphometric characteristics between males and females of mud crab genus *Scylla*. Measured morphometric characteristics include carapace width (CW), CW at spine 8 (8CW), internal carapace width (ICW), carapace length (CL), abdomen width (AW), right cheliped’s dactyl length (DL), right cheliped’s propodus depth (PD), right cheliped’s propodus width (PW), right chelipeds propodus length (PL) and right cheliped’s merus length (ML).

	*S. olivacea* (x¯ ± s.d.)				*S. paramamosain* (x¯ ± s.d.)				*S. tranquebarica* (x¯ ± s.d.)			
	Male (*n* = 900)		Female (*n* = 900)	*t*	Male (*n* = 900)		Female (*n* = 900)	*t*	Male (*n* = 900)		Female (*n* = 900)	*t*
CW	111.84 ± 13.61^*^	>	108.10 ± 10.30^*^	6.58	111.48 ± 11.84^*^	>	109.71 ± 11.41^*^	5.95	115.02 ± 14.00^*^	>	111.06 ± 11.34^*^	3.23
ICW	104.25 ± 12.50^*^	>	101.62 ± 9.31^*^	5.06	103.72 ± 11.13^*^	>	102.07 ± 11.01^*^	6.59	107.39 ± 13.22^*^	>	104.05 ± 10.46^*^	3.17
8CW	112.92 ± 13.74^*^	>	108.51 ± 10.35^*^	7.69	109.91 ± 11.73^*^	>	108.21 ± 11.25^*^	7.16	113.55 ± 14.00^*^	>	109.28 ± 11.14^*^	3.14
CL	77.81 ± 9.08^*^	>	71.91 ± 7.27^*^	15.22	72.16 ± 7.88^*^	>	70.02 ± 7.55^*^	9.02	75.20 ± 9.28^*^	>	71.56 ± 7.74^*^	5.89
AW/CW	0.26 ± 0.01^**^	<	0.44 ± 0.03^**^	−166.19	0.25 ± 0.01^**^	<	0.42 ± 0.02^**^	−229.03	0.25 ± 0.01^**^	<	0.42 ± 0.02^**^	−232.95
DL/CW	1.46 ± 1.8E^−4^**	>	0.71 ± 0.06^**^	409.72	1.36 ± 0.08^**^	>	0.71 ± 0.04^**^	231.12	1.38 ± 0.10^**^	>	0.71 ± 0.04^**^	188.92
PD/CW	0.87 ± 0.9E^−4^**	>	0.78 ± 0.06^**^	44.34	1.01 ± 0.06^**^	>	0.80 ± 0.04^**^	93.25	0.88 ± 0.06^**^	>	0.80 ± 0.05^**^	34.68
PL/CW	2.39 ± 2.0E^−4^**	<	2.55 ± 0.20^**^	−23.85	2.18 ± 0.12^**^	<	2.57 ± 0.13^**^	−69.27	2.56 ± 0.17^**^	<	2.58 ± 0.15^**^	−2.80
ML/CW	0.57 ± 0.4E^−4^**	>	0.56 ± 0.04^**^	5.45	0.54 ± 0.03^**^	<	0.59 ± 0.03^**^	−37.76	0.52 ± 0.03^**^	<	0.59 ± 0.35^**^	−43.23
PW/CW	0.50 ± 0.7E^−4^**	>	0.49 ± 0.04^**^	9.56	0.50 ± 0.03^**^	>	0.41 ± 0.02^**^	80.79	0.46 ± 0.03^**^	>	0.41 ± 0.02^**^	41.98

**Notes.**

* Significant difference (*P* < 0.01)The insertion was also identified as STR expansion.

** Significant difference (*P* < 0.001) between sexes within a species.

x¯ mean s.d. standard deviation *n* number of measured individuals *t* *t*-value

Based on the discriminant function analysis, all morphometric ratios of *S. olivacea*, *S. tranquebarica* (except ML/CW) and *S. paramamosain* (except 8CW/CW) showed significant variation between sexes. The discriminant functions of all three species are listed in Table 2. All derived functions explaining 100% of the variance had a small lambda (0.012–0.022) and were significant, with *P* values of less than 0.001 (Table S1). All three functions correctly classified 100% of the 1,800 adults of known sex and had a cross-validation percentage of 100%. The cut-off values of these functions were 0, with >0 = male in *S. olivacea* and *S. paramamosain* and >0 = female in *S. tranquebarica*.

**Table 2:** Discriminant function of morphometric ratios and correct classification of males and females of each *Scylla* spp. Measured morphometric characteristics include carapace width (CW), CW at spine 8 (8CW), internal carapace width (ICW), carapace length (CL), abdomen width (AW), right cheliped’s dactyl length (DL), right chelipeds propodus depth (PD), right cheliped’s propodus width (PW), right cheliped’s propodus length (PL) and right cheliped’s merus length (ML).

Discriminant function	Correct classification, cross-validation (%)
*Scylla olivacea*	100, 100
$0.048 \frac{8\mathrm{CW}}{\mathrm{CW}} -0.071 \frac{\mathrm{ICW}}{\mathrm{CW}} -0.404 \frac{\mathrm{CL}}{\mathrm{CW}} -1.006 \frac{\mathrm{AW}}{\mathrm{CW}} +0.164 \frac{\mathrm{DL}}{\mathrm{CW}} +0.472 \frac{\mathrm{PD}}{\mathrm{CW}} +0.224 \frac{\mathrm{PL}}{\mathrm{CW}} +0.259 \frac{\mathrm{ML}}{\mathrm{CW}} +0.334 \frac{\mathrm{PW}}{\mathrm{CW}} $	
Cut-off value = 0 (>0 = male; <0 = female)	
*S. tranquebarica*	100, 100
$0.079 \frac{\mathrm{ICW}}{\mathrm{CW}} -0.152 \frac{8\mathrm{CW}}{\mathrm{CW}} +0.092 \frac{\mathrm{CL}}{\mathrm{CW}} +0.926 \frac{\mathrm{AW}}{\mathrm{CW}} -0.068 \frac{\mathrm{DL}}{\mathrm{CW}} -0.234 \frac{\mathrm{PD}}{\mathrm{CW}} -0.249 \frac{\mathrm{PL}}{\mathrm{CW}} -0.254 \frac{\mathrm{PW}}{\mathrm{CW}} $	
Cut-off value = 0 (>0 = female; <0 = male)	
*S. paramamosain*	100, 100
$0.046 \frac{\mathrm{ICW}}{\mathrm{CW}} -0.074 \frac{\mathrm{CL}}{\mathrm{CW}} -0.948 \frac{\mathrm{AW}}{\mathrm{CW}} +0.099 \frac{\mathrm{DL}}{\mathrm{CW}} +0.496 \frac{\mathrm{PD}}{\mathrm{CW}} +0.241 \frac{\mathrm{PL}}{\mathrm{CW}} +0.05 \frac{\mathrm{ML}}{\mathrm{CW}} +0.41 \frac{\mathrm{PW}}{\mathrm{CW}} $	
Cut-off value = 0 (>0 = male; <0 = female)	

**Figure 2:**
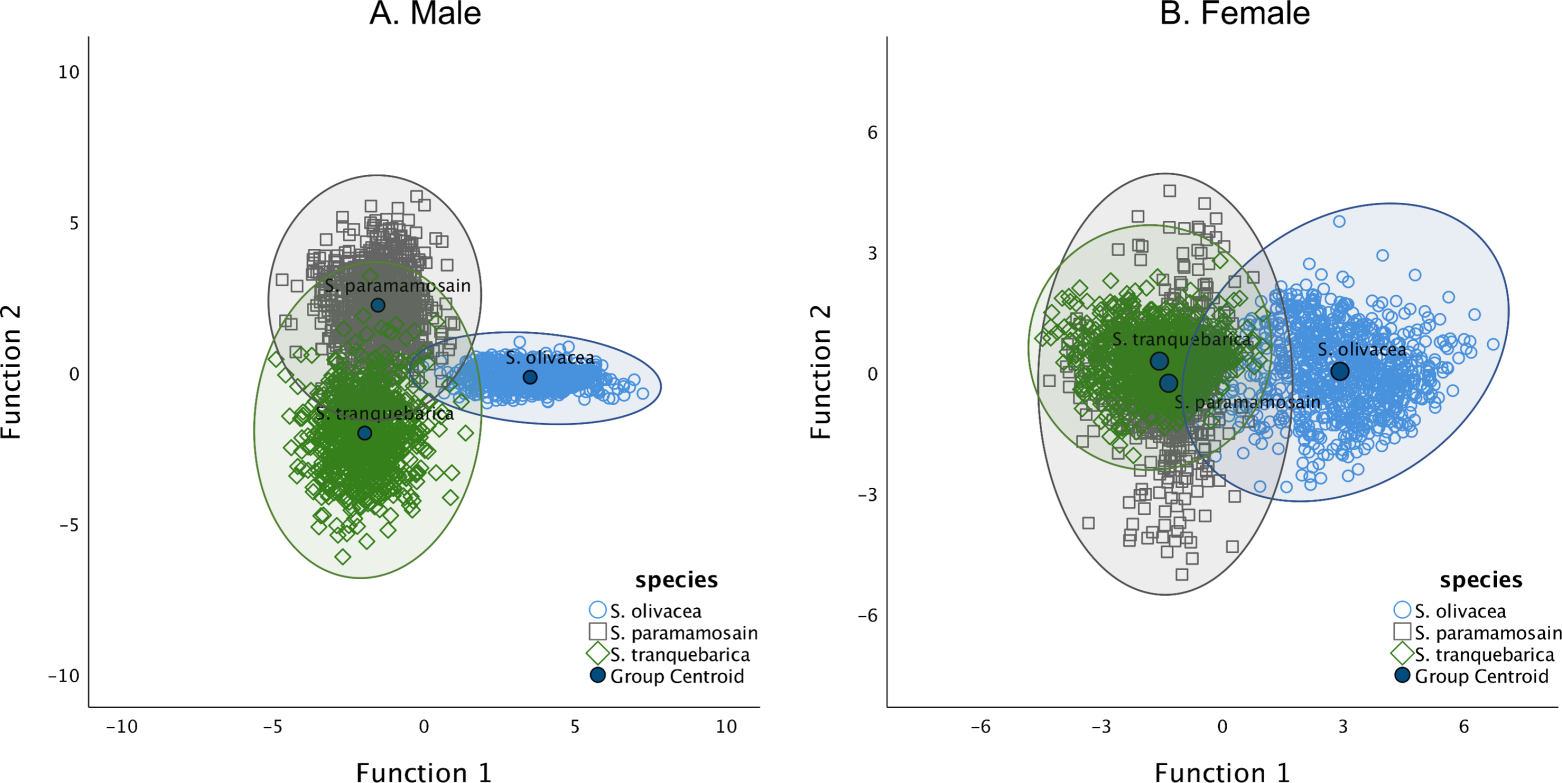
The grouping of *Scylla olivacea, S. paramamosain* and *S. tranquebarica* based on the two discriminant functions. The two axes determine the canonical space of the discriminant analysis for (A) males and (B) females of three *Scylla* species. Group envelopes (ellipses) are centered on the group centroids.

By analysing the nine morphometric ratios of males and females using DFA, we found that all ratios were significantly important in discerning males whereas all except PD/CW were essential in discerning females of the three *Scylla* species (Table S1). The derived functions correctly classified 97.4% of the original grouped cases and 97.3% of cross-validated grouped cases in males (Fig. 2, Table 3). The highest species with correct classification was *S. olivacea* (99.7%). In females, 74.0% of the original grouped cases were correctly classified (73.6% in cross-validated cases) (Fig. 2, Table 3). Unlike in males where all species had a correct classification percentage of above 94%, that of females was high only for *S. olivacea* (95.3%). The correct classification percentage was lower than 65% for females of *S. tranquebarica* (62.2%) and *S. paramamosain* (63.4%). Further, almost all of the misclassified females were between *S. tranquebarica* and *S. paramamosain*. All morphometric ratios were significantly different in males and females of *Scylla* (Table S2). Overall, the secondary sexual characteristics and body size dimensions in relation to CW of *S. olivacea* were greater than that of *S. tranquebarica* and *S. paramamosain* in both sexes. The CW of *S. olivacea* was significantly smaller than its 8CW in both males (*t*_899_ =  − 29.14; *P* < 0.001) and females (*t*_899_ =  − 16.01; *P* < 0.001) whereas the opposite, i.e., CW larger than 8CW, was observed in both sexes of *S. paramamosain* and *S. tranquebarica* (*S. paramamosain* male: *t*_899_ = 117.27; *P* < 0.001; *S. paramamosain* female: *t*_899_ = 83.91; *P* < 0.001; *S. tranquebarica* male: *t*_899_ = 89.53; *P* < 0.001; *S. tranquebarica* female: *t*_899_ = 82.81; *P* < 0.001).

**Table 3:** Classification results of males and females of *Scylla* species.

Sex			Predicted Group Membership, n (%)			Total, *n* (%)	Correct classification, cross-validation (%)
		Species	*S. olivacea*	*S. paramamosain*	*S. tranquebarica*		
Male	Original group	*S. olivacea*	897 (99.7)	3 (0.3)	0 (0.0)	900 (100)	97.4, 97.3
				*S. paramamosain*	1 (0.1)	879 (97.7)		20 (2.2)	900 (100)	
			*S. tranquebarica*	3 (0.3)	43 (4.8)	854 (94.9)		900 (100)	
		Cross-validated	*S. olivacea*	897 (99.7)	3 (0.3)	0 (0.0)		900 (100)	
				*S. paramamosain*	1 (0.1)	878 (97.6)		21 (2.3)	900 (100)	
	*S. tranquebarica*		3 (0.3)	44 (4.9)	853 (94.8)	900 (100)	
Female	Original group	*S. olivacea*	858 (95.3)	36 (4.0)	6 (0.7)	900 (100)	74.0, 73.6
				*S. paramamosain*	0 (0.0)	578 (64.2)		322 (35.8)	900 (100)	
			*S. tranquebarica*	6 (0.7)	333 (37.0)	561 (62.3)		900 (100)	
		Cross-validated	*S. olivacea*	857 (95.2)	37 (4.1)	6 (0.7)		900 (100)	
				*S. paramamosain*	0 (0.0)	571 (63.4)		329 (36.6)	900 (100)	
	*S. tranquebarica*		6 (0.7)	334 (37.1)	560 (62.2)	900 (100)	

## Discussion

Within the infraorder Brachyura, males often exhibit larger body size dimensions than females in most species, including the three species of the genus *Scylla* as shown in this study. The larger body size of males may be linked to the role it plays during the mating process (Hartnoll, 1969). Mating in portunid crabs, including in *Scylla* spp., involves an intermoult male and a newly-moulted female (Waiho et al., 2015). After mating, the male often engaged in post-copulatory guarding to safeguard the newly-mated female while her carapace hardens (Waiho et al., 2015). In a recent assortative mating experiment conducted on three *Scylla* species (*S. olivacea, S. tranquebarica* and *S. paramamosain*), all males in mating pairs were larger than their female counterparts (Fazhan et al., 2017b). It is postulated that smaller females might be easier for males to cradle during pre-copulatory and post-copulatory guarding periods, and facilitate easier copulation as males need to flip over the females in order to copulate. Thus, the results of our study on three species of the genus *Scylla* are in accordance with the notion that Portunids follow the classic sexual dimorphism pattern, with males larger than females (Doi et al., 2008; Nordhaus, Diele & Wolff, 2009).

In addition to body size dimensions, sexual dimorphism in the secondary sexual characteristics of *Scylla* is prominent during the adult stage. Changes in the overall dimension of AW in *Scylla* females once they reach sexual maturity, i.e., become more globular and dark in color, are expected and has been used as a key factor to determine size at sexual maturity in wild populations of *Scylla* spp. (Overton & Macintosh, 2002; Waiho et al., 2016) and other brachyuran species (Mura, Orrù & Cau, 2005; Williner et al., 2014; Öndes, Kaiser & Murray, 2017). Unlike females, the AW of males retains almost the same colouration and shape since juvenile stages (Waiho, Fazhan & Ikhwanuddin, 2016). Variation in AW between sexes is common in most brachyuran species (Haefner Jr, 1990; Josileen, 2011; Davanso et al., 2016; Öndes, Kaiser & Murray, 2017). The broadening of the abdomen in *Scylla* females occurs when they reach sexual maturity (Waiho et al., 2016) and is linked with reproductive functions such as attachment of eggs and incubation. Wider abdomens in females often indicate larger egg mass, thus capable of producing more larvae (Crawford & De Smidt, 1922). In contrast, the shape of the abdomen in males maintains as they grow since its main function is to protect the copulatory organ called the gonopods (Hartnoll, 1982).

For most cheliped dimensions, males of all three *Scylla* spp. showed significantly larger values than females. Similar male-biased dimorphism in cheliped dimensions was also reported in other decapod crustaceans, such as lobsters (Claverie & Smith, 2010; Lezcano et al., 2015) and shrimps (Mashiko, 1981; Mantel & Dudgeon, 2005). In *Scylla*, apart from body size dimensions, cheliped size is also postulated to play a significant role in female courting and settling a fight during mate guarding (Waiho et al., 2015). Unlike females, males are required to fight and fence off rivals during copulatory guarding. Thus, the larger cheliped size of males is advantageous, both as a courting aid and also a fighting tool.

All measured morphological characteristics showed similar patterns in all three *Scylla* species, except for 8CW. When compared with CW, the 8CW of *S. olivacea* was similar or greater than its CW in both males and females. In contrast, the CWs of *S. paramamosain* and *S. tranquebarica* were always greater than their 8CWs. The evolution of body sizes is often species-related and the divergence of specific body forms may be linked to their different habitat and ecology (Overton & Macintosh, 2002). Based on the results of this study, we postulate that the smaller CW compared to 8CW in *S. olivacea* might be attributed to the microhabitat they live in. *Scylla olivacea* is known to inhabit mangrove forests at the upper intertidal zones (Fazhan et al., 2017a). Therefore, their less spiny features (Overton & Macintosh, 2002), and smaller CW (body size) probably allow easier burrowing into the substrate and movement within the mangrove root system. On the other hand, *S. paramamosain* and *S. tranquebarica* are found in estuaries and subtidal zones (Keenan, Davie & Mann, 1998; Fazhan et al., 2017a). The pronounced body spines, including larger body size or CW over 8CW of *S. paramamosain* and *S. tranquebarica* perhaps function as weapons to protect them against predators in the open waters.

Inter-species variation in all measured morphometric ratios was significant, especially in males as the discriminant functions incorporating all morphometric ratios could correctly differentiate among species up to 94%. In females, however, the discriminant functions could correctly classify only the *S. olivacea* species to a high rate (95.3%) while the correct classification percentage for the females of the other two species were below 65%. This strongly indicates that morphometric characteristics varied greatly only among males of these three *Scylla* species, but less prominent between the females of *S. paramamosain* and *S. tranquebarica*. Cheliped dimensions of brachyuran crabs are often male-biased and significant variation exists among males of different populations or congeneric males (Lezcano et al., 2012; Kalate et al., 2018). However, unlike males, the sexual dimorphic traits in brachyuran females such as cheliped dimensions are more subtle, especially among congenerics (Silva, Mesquita & Paula, 2010). The inclusion of other measurable body traits in the future, such as frontal lobe and sternum measurements (Keenan, Davie & Mann, 1998; Fazhan et al., 2020), might aid in distinguishing between females of *S. paramamosain* and *S. tranquebarica*. Another possible reason for the lack of variation between the females of *S. paramamosain* and *S. tranquebarica* is the method employed in this study, i.e., traditional morphometric technique. Kalate et al. (2018) demonstrated that the carapace features of freshwater crab *Potamon elbursi* Pretzmann, 1962 females among two populations were significantly distinct via geometric morphometry—a geometric method based on the multivariate space of biological shapes (Thompson & Bonner, 1917), but not traditional morphometry. Future research could employ both traditional morphometric and geometry morphometric approaches in attempt to identify morphometric traits that might be useful in discerning the females of congeneric *Scylla* species. Finally, various factors within an ecological niche, such as food availability, water depth and temperature are known to influence morphometric variations of crabs (Giri & Anna, 2008; Torres, Collins & Giri, 2014; Kalate et al., 2018). Thus, the similarities between females of *S. paramamosain* and *S. tranquebarica* could also be attributed to their adaptation to the same microhabitats—estuarines—whereas that of *S. olivacea* prefer upper river mouths with lower salinity (Fazhan et al., 2020; Fazhan et al., 2021).

All morphometric characteristics contribute significantly to the discriminant models of all three *Scylla* species, except for ML (for *S. tranquebarica*) and 8CW (for *S. paramamosain*). This indicates that all measured morphometric characteristics exhibited gender dimorphism, although not as pronounced in the ML of *S. tranquebarica* and the 8CW of *S. paramamosain*. Discriminant models have been widely used in the differentiation and selection of livestock (Leotta, 2004; Purzyc, Kobryńczuk & Bojarski, 2011; Assamoah-Boaheng & Sam, 2016) and fish stock (Cadrin, 2000; Khemiri et al., 2018). The discriminant functions made available in this study would be useful in assessing, managing and conserving different mud crab populations that are living in sympatry. Further, phenotypically pure local genetic resources would be easily selected for future breeding improvement purposes.

## Conclusions

The morphometric results of the present study show that distinct morphometric variation was observed between males and females of *Scylla* spp. and highlight the influence of sexual dimorphism on their external morphologies. The morphometric variations of the secondary sexual characteristics between sexes are associated with reproduction and reveal the different functions of the same body structure in males and females of *Scylla* spp. The similarity in sexually dimorphic traits among *Scylla* species further supports the influence of sexual selection on the development of secondary sexual characteristics in this genus. Based on the results of DFA, the significant morphometric variations among congeneric males of the three *Scylla* species, but less prominent variations among the females of *S. paramamosain* and *S. tranquebarica* imply that morphological variations in *Scylla* might be influenced by both gender and environmental conditions. Future comparative behavioural studies could elucidate the adaptive functions of secondary sexual characteristics between sexes among species of this genus.

##  Supplemental Information

10.7717/peerj.10936/supp-1 Data S1 Measurement data for Scylla olivacea, S. tranquebarica and S. paramamosain

10.7717/peerj.10936/supp-2 Table S1 The eigenvalues and Wilks’ lamda test for the derived discriminant functions of three *Scylla* species

10.7717/peerj.10936/supp-3 Table S2 One-Way ANOVA and Games-Howell test in males and females of *Scylla*

## Additional Information and Declarations

### Data Availability

The following information was supplied regarding data availability: All raw measurements are available in the Supplemental Files.
