# Supplementary material for: Sexual dimorphism in mud crabs: a tale of three sympatric Scylla species
Source: PeerJ. 2021 Apr 12;9:e10936. doi: 10.7717/peerj.10936 (PMC8048398; doi:10.7717/peerj.10936)
Supplement: Table S1 [file peerj-09-10936-s002.docx]

Supplementary Table 1. The eigenvalues and Wilks’ lamda test for the derived discriminant functions of three *Scylla* species.

| Species | Function | Eigenvalues | | | | Wilk’s lamda | | | |
| --- | --- | --- | --- | --- | --- | --- | --- | --- | --- |
|  |  | Eigenvalue | % of Variance | Cummulative % | Cannonical correlation | Wilk’s lamda | Chi-square | df | *P* value |
| *S. olivacea* | 1 | 56.56 | 100 | 100 | 0.99 | 0.017 | 7268.66 | 9 | < 0.001 |
| *S. tranquebarica* | 1 | 44.11 | 100 | 100 | 0.99 | 0.022 | 6833.37 | 8 | < 0.001 |
| *S. paramamosain* | 1 | 85.46 | 100 | 100 | 0.99 | 0.012 | 8000.62 | 8 | < 0.001 |
| Combined males | 1 | 6.18 | 67.3 | 67.3 | 0.93 | 0.035 | 9045.27 | 18 | < 0.001 |
|  | 2 | 3.01 | 32.7 | 100 | 0.87 | 0.250 | 3738.23 | 8 | < 0.001 |
| Combined females | 1 | 4.26 | 98.8 | 98.8 | 0.90 | 0.181 | 4600.51 | 16 | < 0.001 |
|  | 2 | 0.05 | 1.2 | 100 | 0.22 | 0.952 | 131.46 | 7 | < 0.001 |
