# Supplementary material for: Sexual dimorphism in mud crabs: a tale of three sympatric Scylla species
Source: PeerJ. 2021 Apr 12;9:e10936. doi: 10.7717/peerj.10936 (PMC8048398; doi:10.7717/peerj.10936)
Supplement: Table S2 [file peerj-09-10936-s003.docx]

Supplementary Table 2. One-Way ANOVA and Games-Howell test in males and females of *Scylla*. Measured morphometric characteristics include carapace width (CW), CW at spine 8 (8CW), internal carapace width (ICW), carapace length (CL), abdomen width (AW), right cheliped’s dactyl length (DL), right cheliped’s propodus depth (PD), right cheliped’s propodus width (PW), right cheliped’s propodus length (PL) and right cheliped’s merus length (ML).

| Morphometric ratio | Sex | One-Way ANOVA | Games-Howell (‘>’ indicates *P* < 0.05, ‘=’ indicates *P* ≥ 0.05) |
| --- | --- | --- | --- |
| ICW/CW | Male | *F*_2,1783.46_ = 34.813, *P* < 0.001 | ST > SO > SP |
|  | Female | *F*_2,1625.18_ = 62.210, *P* < 0.001 | SO > ST > SP |
| 8CW/CW | Male | *F*_2,1654.86_ = 2342.448, *P* < 0.001 | SO > ST > SP |
|  | Female | *F*_2,1732.75_ = 2377.821, *P* < 0.001 | SO > SP > ST |
| CL/CW | Male | *F*_2,1783.63_ = 585.439, *P* < 0.001 | SO > ST > SP |
|  | Female | *F*_2,1748.54_ = 137.902, *P* < 0.001 | SO > ST > SP |
| AW/CW | Male | *F*_2,1759.05_ = 358.257, *P* < 0.001 | SO > SP > ST |
|  | Female | *F*_2,1748.02_ = 137.761, *P* < 0.001 | SO > ST > SP |
| DL/CW | Male | *F*_2,1779.06_ = 1479.049, *P* < 0.001 | SO > SP = ST |
|  | Female | *F*_2,1735.79_ = 75.619, *P* < 0.001 | SO > ST > SP |
| PD/CW | Male | *F*_2,1794.00_ = 1927.478, *P* < 0.001 | SP > SO > ST |
|  | Female | *F*_2,1750.14_ = 10.276, *P* < 0.001 | SO = ST > SP |
| PL/CW | Male | *F*_2,1782.75_ = 378.210, *P* < 0.001 | SO > ST > SP |
|  | Female | *F*_2,1740.02_ = 5.631, *P* < 0.001 | ST > SO, SP = SO, SP = ST |
| ML/CW | Male | *F*_2,1787.34_ = 1622.788, *P* < 0.001 | SO > SP > ST |
|  | Female | *F*_2,1759.32_ = 403.167, *P* = 0.004 | SP = ST > SO |
| PW/CW | Male | *F*_2,1785.52_ = 3397.022, *P* < 0.001 | SO > SP > ST |
|  | Female | *F*_2,1733.07_ = 893.802, *P* < 0.001 | SO > ST > SP |

Note: SO = *S. olivacea*; ST = *S. tranquebarica*; SP = *S. paramamosain*; *F* = *F* value; *P* = *P* value.
